# The Dysfunctional Immune System in Common Variable Immunodeficiency Increases the Susceptibility to Gastric Cancer

**DOI:** 10.3390/cells9061498

**Published:** 2020-06-19

**Authors:** Irene Gullo, Catarina Costa, Susana L. Silva, Cristina Ferreira, Adriana Motta, Sara P. Silva, Rúben Duarte Ferreira, Pedro Rosmaninho, Emília Faria, José Torres da Costa, Rita Câmara, Gilza Gonçalves, João Santos-Antunes, Carla Oliveira, José C. Machado, Fátima Carneiro, Ana E. Sousa

**Affiliations:** 1 Department of Pathology, Centro Hospitalar Universitário de São João (CHUSJ), 4200-319 Porto, Portugal; igullo@med.up.pt (I.G.); catarinapedrosacosta@gmail.com (C.C.); gilzasofia@gmail.com (G.G.); 2 Department of Pathology, Faculty of Medicine of the University of Porto (FMUP), 4200-319 Porto, Portugal; carlaol@i3s.up.pt (C.O.); josem@ipatimup.pt (J.C.M.); 3 Institute of Molecular Pathology and Immunology, University of Porto (Ipatimup), 4200-135 Porto, Portugal; 4 Instituto de Investigação e Inovação em Saúde (i3S), University of Porto, 4200-135 Porto, Portugal; 5 Instituto de Medicina Molecular João Lobo Antunes, Faculdade de Medicina, Universidade de Lisboa, 1649-028 Lisbon, Portugal; cristina.ferreira@chln.min-saude.pt (C.F.); adrianar@campos.ul.pt (A.M.); sara.silva@chln.min-saude.pt (S.P.S.); rduarteferreira@medicina.ulisboa.pt (R.D.F.); pedro.rosmaninho@medicina.ulisboa.pt (P.R.); asousa@medicina.ulisboa.pt (A.E.S.); 6 Centro de Imunodeficiências Primárias do Centro Académico de Medicina de Lisboa, 1649-028 Lisbon, Portugal; 7 Centro Hospitalar Universitário Lisboa Norte, 1600-190 Lisbon, Portugal; 8 Serviço de Imunoalergologia, Centro Hospitalar Hospitalar e Universitário de Coimbra, 3004-561 Coimbra, Portugal; emiliamfaria@chuc.min-saude.pt; 9 Serviço de Imunoalergologia, Centro Hospitalar Hospitalar Universitário de São João (CHUSJ), 4200-319 Porto, Portugal; zecatoco@sapo.pt; 10 Serviço de Imunoalergologia, Hospital Dr Nélio Mendonça, 9000-177 Funchal, Portugal; ritacamara@sesaram.pt; 11 Department of Gastroenterology, Centro Hospitalar Universitário de São João (CHUSJ), 4200-319 Porto, Portugal; joao.claudio.antunes@gmail.com

**Keywords:** gastric cancer, common variable immunodeficiency, immune microenvironment, *Helicobacter pylori*, lymphocytic gastritis, immune dysfunctionality, inborn errors of immunity

## Abstract

Gastric carcinoma (GC) represents the most common cause of death in patients with common variable immunodeficiency (CVID). However, a limited number of cases have been characterised so far. In this study, we analysed the clinical features, bacterial/viral infections, detailed morphology and immune microenvironment of nine CVID patients with GC. The study of the immune microenvironment included automated digital counts of CD20+, CD4+, CD8+, FOXP3+, GATA3+ and CD138+ immune cells, as well as the evaluation of PD-L1 expression. Twenty-one GCs from non-CVID patients were used as a control group. GC in CVID patients was diagnosed mostly at early-stage (*n* = 6/9; 66.7%) and at younger age (median-age: 43y), when compared to non-CVID patients (*p* < 0.001). GC pathogenesis was closely related to *Helicobacter pylori* infection (*n* = 8/9; 88.9%), but not to Epstein-Barr virus (0.0%) or cytomegalovirus infection (0.0%). Non-neoplastic mucosa (non-NM) in CVID-patients displayed prominent lymphocytic gastritis (100%) and a dysfunctional immune microenvironment, characterised by higher rates of CD4+/CD8+/Foxp3+/GATA3+/PD-L1+ immune cells and the expected paucity of CD20+ B-lymphocytes and CD138+ plasma cells, when compared to non-CVID patients (*p* < 0.05). Changes in the immune microenvironment between non-NM and GC were not equivalent in CVID and non-CVID patients, reflecting the relevance of immune dysfunction for gastric carcinogenesis and GC progression in the CVID population.

## 1. Introduction

Inborn errors of immunity comprise an expanding group of more than 400 diseases which have provided a remarkable unexplored context for research [1]. Common variable immunodeficiency (CVID) is the most common clinically relevant primary immunodeficiency [2]. It is defined by defects in B-cell differentiation into memory B cells and Ig-secreting plasma cells [2,3,4], though phenotypic and functional abnormalities have been increasingly recognised also in T cells [5,6] and innate immunity [7,8].

Increasing evidence points to a continuous spectrum of immunological disturbances, leading to an ongoing debate on the CVID diagnostic criteria [9]. Extreme depletion of the naïve CD4 compartment (naïve CD4 T cells below 10%) has been considered the most sensitive indicator to suggest some cellular immunodeficiency in patients with CVID, which is associated with higher prevalence of severe non-infectious complications [10]. In addition to recurrent infections primarily in the respiratory and gastrointestinal tracts, the most typical presentation in CVID, clinical manifestations related to immune dysregulation in these patients include autoimmune disorders, a variable spectrum of lymphoproliferative diseases and malignancies [11,12].

Patients with CVID bear a 10 to 47-fold increased risk for developing cancer in comparison with the general population [2,13,14,15]. Based on a recent meta-analysis, the overall prevalence of malignancy in CVID patients is 8.6% [16], the most common encompassing lymphoma (40.5%) and gastric adenocarcinoma (7.5%), followed at a much lower frequency by breast, skin, thymic cancer, leukaemia and melanoma, among others [16].

Gastric cancer ranks second among CVID-related cancers, after the expected lymphoproliferative diseases, and represents the first cause of cancer-related death in this population [17]. In contrast, according to the most recent GLOBOCAN estimates, gastric cancer is the fifth most frequently diagnosed malignancy and the third leading cause of cancer-related death worldwide in the general population [18].

In spite of the high incidence of gastric cancer in CVID patients, only 59 cases have been reported to date in a meta-analysis of 48 studies with a total of 8123 CVID patients [16], and detailed clinicopathological characterisation is limited to solely two case series [17,19].

Patients with gastric cancer and CVID are usually 15 years younger than those without CVID [20]. CVID-associated gastric cancers are frequently intestinal-type moderately to poorly differentiated adenocarcinomas with high numbers of intra-tumoral lymphocytes [19]. Malignancy generally develops on a background of severe atrophic metaplastic pangastritis [19,20].

The high incidence of gastric adenocarcinoma in CVID patients has been linked to genetic predisposition, persistent mucosal inflammation and decreased clearance of oncogenic viral and bacterial infections, including *Helicobacter pylori* infection [20,21]. Autoimmune processes, as in the case of pernicious anaemia, are also known to increase the risk for gastric cancer in CVID, as reported in the general population [22]. It is plausible that impairment of tumour cell immune surveillance may facilitate survival and proliferation of pre-malignant cells. Despite the important influence that the immune microenvironment may exert in CVID-associated gastric cancer pathogenesis and progression, an in-depth analysis of the immune landscape in this context is still missing. Moreover, patients with inborn errors of immunity may provide a unique, not yet explored, context to decipher the interplay of immunological and environmental factors in gastric adenocarcinoma.

In this study, we performed a detailed clinical, histopathological and immunophenotypic analysis of CVID-associated gastric cancer and background non-neoplastic mucosa, aiming at uncovering biological features that may be associated with increased susceptibility of CVID patients to gastric cancer. In particular, to elucidate mechanisms that could mediate gastric cancer pathogenesis and progression in CVID patients, we explored the putative influence of bacterial and viral infections as well as the quality, density and spatial/temporal distribution of the immune microenvironment. This represents the first attempt to provide a detailed description of the disease.

## 2. Materials and Methods

### 2.1. Patient Series

Nine gastric adenocarcinomas from formalin fixed paraffin-embedded (FFPE) gastric cancer samples of CVID patients, including biopsies (*n* = 2) and surgical specimens (*n* = 7), were obtained from a cohort of CVID patients under follow-up at Centro de Imunodeficiências Primárias (CIDP) of Centro Académico de Medicina de Lisboa (Lisbon, Portugal) (*n* = 6) and from other hospitals belonging to the national network of CIDP (*n* = 3).

CVID was diagnosed based on marked decrease of IgG (at least two standard deviations below the mean for age) and a marked decrease in at least one of the isotypes IgM or IgA, impaired response to vaccines and exclusion of secondary causes for hypogammaglobulinemia [2]. Twenty-one gastric cancer FFPE samples from non-CVID patients with early-stage gastric cancer were also obtained from the Departments of Pathology and of Gastroenterology at Centro Hospitalar Universitário São João (CHUSJ) and used as control group.

Clinical features and medical records, collected from the files of CIDP and CHUSJ, were analysed, including gender, age, relevant personal and family history and treatment for gastric cancer. Data from stool cultures and PCR analysis relative to gastrointestinal infections, routinely performed in CVID patients, were also retrieved.

Histopathological characterisation of gastric adenocarcinomas and the background non-neoplastic mucosa, distant from and adjacent to gastric cancer (when available), was performed on haematoxylin and eosin (H&E)-stained slides. Particularly, in non-neoplastic mucosa the presence of lymphocytic gastritis, atrophic gastritis with or without neutrophilic activity, lymphoid aggregates and intestinal metaplasia (IM) was evaluated. Moreover, the presence of precursor lesions was specified. The tumour histotype was defined according to the classifications of Laurén [23] and the World Health Organization (WHO) (2019) [24]. Tumour staging was evaluated according to the 2018 AJCC staging system [25].

The study was conducted upon the approval of the Ethical Board of the Centro Hospitalar Universitário Lisboa Norte (CHULN) and of the Faculdade de Medicina da Universidade de Lisboa (FMUL).

### 2.2. Immunohistochemistry (IHC) and Epstein-Barr Virus (EBV) In Situ Hybridization (ISH)

Serial 3-μm sections were prepared from one representative FFPE block. IHC staining was performed in all CVID and non-CVID gastric cancer cases with antibodies against CMV (clone CCH2 and DDG9, 1:1000; DAKO), CD20, (clone L26, prediluted; Ventana Medical Systems), CD8 (clone SP57, prediluted; Ventana Medical Systems), CD4 (clone SP35, prediluted; Ventana Medical Systems), GATA3 (clone D13C9, 1:200; Cell Signaling Technology), Foxp3 (clone AB54501, 1:100; abcam), CD138/Syndecan-1 (clone B-A38, prediluted; Cell Marque) and PD-L1 (clone 22C3, 1:80; DAKO). Samples were processed in the automatic Ventana Benchmark Ultra platform using an Optiview Universal DAB Detection Kit and an Optiview Amplification Kit for PD-L1 staining. EBV infection was studied using chromogenic ISH for EBV-encoded RNA (EBER-ISH, INFORM EBER probe, Ventana Medical Systems) using the same equipment, with enzymatic digestion (ISH protease) and an iViewBlue detection kit. The detailed protocols are presented in Table S1.

### 2.3. Digital Image Analysis

Full thickness consecutive sections, stained for H&E, CMV, CD20, CD4, CD8, Foxp3, GATA3, CD138/Syndecan-1 and PD-L1 were scanned with a 40× objective, using a Nanozoomer S60 slide scanner (Hamamatsu). QuPath Open source software (0.2.0-m9) was used for digital pathology image analysis [26].

For each case, a representative field at 200× magnification was selected in the distant and adjacent non-neoplastic mucosa (when available), precursor lesions (when present) and gastric cancer. A positive cell count function, which includes automatic segmentation for cell detection and automatic classification of positivity was conducted in the lamina propria/stroma and intraepithelial compartments, properly separated after manually outlining the polygon region of interest.

### 2.4. Evaluation of PD-L1 Expression

Two pathologists (IG, CC) independently evaluated PD-L1 expression, and discordant evaluations were discussed to reach an agreement. PD-L1 immunoexpression was assessed in distant and adjacent non-neoplastic mucosa, precursor lesions and gastric cancer. An overall slide eyeball assessment was conducted in the non-neoplastic mucosa. The presence or absence of PD-L1+ lymphocytes was noted. An estimate of the percentage of PD-L1+ immune cells in the lamina propria (<10% and ≥10%) was given. For the evaluation of precursor lesions and adenocarcinomas, the combined positive score (CPS) was applied as previously described [27].

### 2.5. Statistical Analysis

IBM SPSS (release 23.0.0) and GraphPad Prism 8 were used for statistical analysis. All tests were two-sided, and differences were considered significant when *p* < 0.05. Comparisons of categorical variables were performed using a Chi square test or Fisher’s exact test, as appropriate. For multiple categorical variables, a post-hoc test was used by applying Bonferroni correction. Comparisons of quantitative variables were performed using non-parametric tests, as the distribution of normality was variable within different immune biomarkers. Accordingly, Mann–Whitney and Wilcoxon tests were used, for independent and dependent variables, respectively.

## 3. Results

The series of patients analysed in this study encompasses nine patients diagnosed with CVID and with gastric cancer. Six of the nine CVID patients belong to a cohort of CVID patients previously described [28] and were diagnosed during a thorough follow-up at CIDP (Lisbon, Portugal), which includes a regular esophagogastroduodenoscopy surveillance protocol [22]. During the period 2007–2020, the total number of patients with CVID followed at CIDP was 98, and the six patients who developed gastric cancer accounted for 6.1% of the cohort subjects. Overall, gastric cancer was the most common malignancy diagnosed in this CVID cohort. The remaining three patients were recruited from other hospitals belonging to the national network of CIDP.

### 3.1. Clinicopathological Features of CVID Patients with Gastric Cancer

#### 3.1.1. Clinical Data

Five of the nine gastric cancer cases occurred in women (55.6%). CVID and gastric cancer were diagnosed at the median age of 21 years (range: 15–46) and 43 years (range: 27–62), respectively. The development of gastric cancer occurred within a median period of 19.0 years (range: 6–38) from the CVID diagnosis. Detailed epidemiological and clinicopathological features of CVID patients with gastric cancer are presented in Table S2.

Only one patient presented family history of primary immunodeficiency disorders, namely one brother (patient 5) with selective IgA deficiency. This is the usual pattern in CVID since the majority of the cases have a polygenic cause. Seven CVID patients reported malignancy other than gastric cancer in 15 relatives, including breast, colorectal, prostate, cervical cancer, renal cell carcinoma and central nervous system (CNS) malignancy (Table S2). A family history of gastric cancer was present in two patients (patient 2 and 9), one (patient 2) being diagnosed with diffuse gastric cancer at the age of 43 years. This patient was submitted to *CDH1* and *CTNNA1* genetic testing, which excluded germline causative variants. Two CVID patients were diagnosed with a second malignancy after gastric cancer, namely rectal adenocarcinoma (patient 2) and hepatocellular carcinoma (patient 5).

Six of the nine patients (66.6%) suffered from autoimmune disorders, which included pernicious anaemia (44.4%), autoimmune pancreatitis, rheumatoid arthritis-like symptoms, immune thrombocytopenia, alopecia and psoriasis. Autoimmune disorders were present in four relatives of three patients (type 1 diabetes mellitus, vitiligo, Sjogren’s syndrome and autoimmune thyroid diseases). Four of the nine patients (44.4%) had granulomatous diseases, confirmed using histopathological analysis, namely granulomatous lymphocytic interstitial lung disease (*n* = 4), associated with granulomas affecting skin and CNS in one patient. All patients were receiving IgG replacement therapy when gastric cancer was diagnosed.

Screening for gastrointestinal infection throughout follow-up revealed the presence of *Campylobacter jejuni* (*n* = 5/9; 55.6%), *Salmonella spp* (*n* = 3/9; 33.3%) and *Giardia lamblia* (*n* = 7/9; 77.8%) infection, as well as gut viral infections, including CMV (*n* = 3/9; 33.3%), EBV (*n* = 1/9; 11.1%) and Norovirus (*n* = 1/9; 11.1%).

*H. pylori* infection was searched for in all cases throughout follow-up and eight tested positive (8/9; 88.9%) via a urea breath test and/or histopathology in gastric biopsy samples.

In seven patients, gastrectomy was the primary treatment (total (*n* = 6) or subtotal distal (*n* = 1). During the follow-up period, no tumour recurrence was detected.

Four out of the nine patients died (44.4%) during the study time. Two patients survived less than one year after presenting with unresectable gastric carcinomas (also with hepatocellular carcinoma in one patient) and two patients died from CVID-related complications.

#### 3.1.2. Histopathological Findings in Non-Neoplastic Gastric Mucosa

Table S2 displays the detailed histopathological features in non-neoplastic mucosa for each CVID patient. Abundant mononuclear, predominantly lymphocytic, inflammatory infiltrate in the lamina propria associated with glandular atrophy (chronic atrophic gastritis) was identified in all specimens for which the non-neoplastic gastric mucosa was available (*n* = 7/9). These patients featured a significant decrease in circulating total lymphocyte counts (median: 1240 cells/µL; CI 95%: 480–2220), as compared to healthy individuals (median: 1900 cells/µL; CI 95%: 1700–2300; *p* = 0.0301), suggesting lymphocytic traffic alterations with increased mucosal homing.

In agreement with the diagnosis of CVID, a paucity of plasma cells in the lamina propria was a feature in all cases, as revealed using immunohistochemistry. Non-neoplastic mucosa of two CVID patients disclosed few plasma cells (counts per 20× power field: 22 (patient 4) and 40 (patient 8)). These two CVID patients featured quantifiable frequencies of circulating plasma cells (frequency of CD38^bright^IgM^negative^ within CD19+ cells: 1.1% (patient 4) and 2.2% (patient 8)).

Lymphocytic gastritis, defined by the presence of at least 25 intraepithelial lymphocytes per 100 epithelial cells [29] was a constant feature of the cases analysed (*n* = 7/7; 100.0%). The majority of intraepithelial lymphocytes were CD8+ (median value per 20× power field: 51; CI 95%: 11.1–303.0) and/or GATA3+ (median value per 20× power field: 30.0; CI 95%: 2.1–43.9) (Section 3.3). Two cases showed exuberant lymphocytic gastritis, with an absolute number of CD8+ intraepithelial lymphocytes of 374 and 303 per 20× power field, respectively, in patients 1 and 4. Of note, these patients featured within the lowest counts of circulating CD8 T cells (median count: 405 cells/µL; CI 95%: 125–994; patient 1: 333 cells/µL; patient 4: 125 cells/µL).

**Figure 1:**
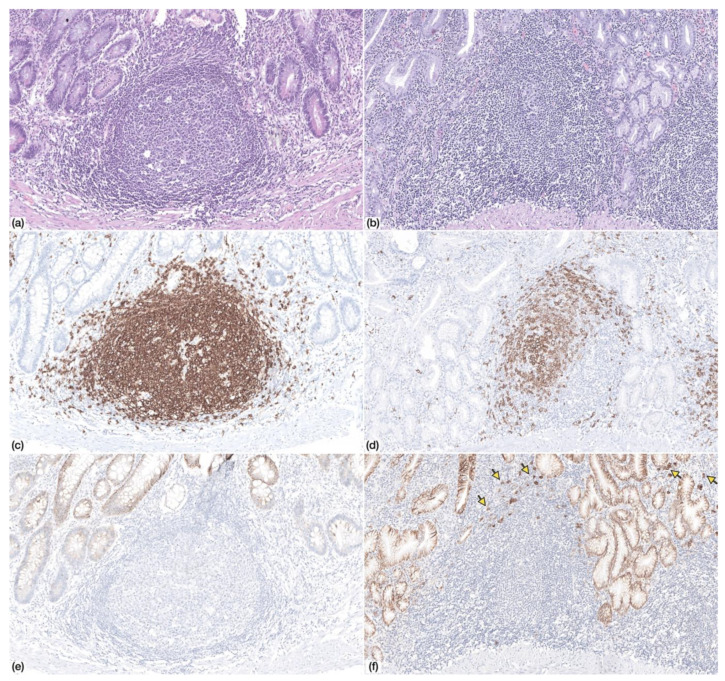
Non-neoplastic gastric mucosa displaying (**a**) lymphoid follicle with germinal center (Haematoxylin and Eosin, HE, 100×) (**c**) with CD20+ lymphocytes (IHC, 100×) and absence of CD138+ plasma cells (**e**) (IHC, 100×)—Patient 9. (**b**) Non-neoplastic gastric mucosa displaying a lymphoid follicle without germinal center (HE, 100×) with (**d**) CD20+ lymphocytes and (**f**) scattered CD138+ plasma cells at the periphery (arrows)—Patient 4.

Multifocal neutrophilic activity was identified in two patients (*n* = 2/7; 28.6%). Chronic atrophic gastritis with IM was identified in all patients (*n* = 7/7; 100%), and in three samples, IM was extensive, occupying almost the totality of the gastric mucosa (*n* = 3/7; 42.9%). Lymphoid follicles were identified in four patients (*n* = 4/7; 57.1%), but germinal centres were only identified in one case (Figure 1).

#### 3.1.3. Histopathological Findings in Gastric Cancer

Five of the nine (*n* = 5/9; 55.6%) gastric cancer cases were localised in the gastric antrum and the remaining four cases (*n* = 4/9; 44.4%) in the corpus.

Pathological staging was available in all gastrectomy specimens and most patients submitted to gastrectomy (*n* = 7/9) were diagnosed at early stages: pT1a (*n* = 4); pT1b (*n* = 2); pT2 (*n* = 1). No lymph node metastases were identified in any gastrectomy specimen.

**Figure 2:**
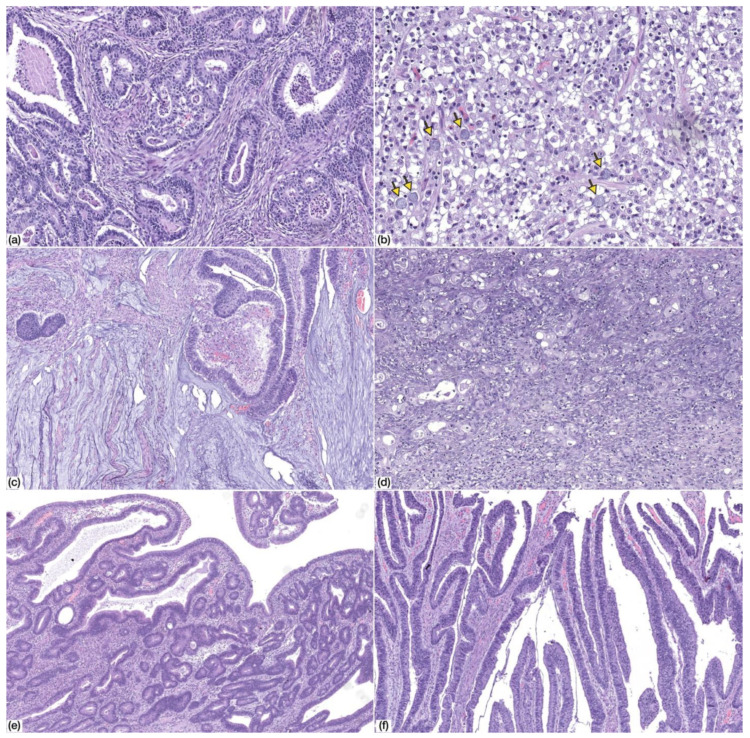
Morphology of common variable immunodeficiency (CVID)-associated gastric cancer. (**a**) Tubular gastric cancer, low-grade (HE, 100× magnification); (**b**) tubular gastric cancer, high-grade (HE, 100× magnification); (**c**) mucinous adenocarcinoma (HE, 50× magnification); (**d**) poorly-cohesive carcinoma, PCC-NOS (HE, 200× magnification). Note the presence of scattered signet ring cells (arrows); (**e,f**) adenomatous lesion, low-grade dysplasia, with tubular (**e**) and villous (**f**) architecture (HE, 50× magnification).

The morphology of gastric cancer developing in the context of CVID was heterogenous (Figure 2). According to the 2019 WHO Classification of Digestive System Tumours [24], the majority of the cases were tubular and/or papillary adenocarcinomas (*n* = 6/9; 66.7%), both low-grade (*n* = 3/6; 50.0%) and high-grade (*n* = 3/6; 50%). The remaining cases included two mucinous adenocarcinomas (*n* = 2/9; 22.2%) and one poorly cohesive carcinoma (*n* = 1/9; 11.1%) of the non-signet ring cell type (PCC-NOS), although scattered signet ring cells were identified.

Precursor adenomatous lesions were identified in two cases and consisted of intestinal type adenoma with low grade dysplasia (Figure 2).

#### 3.1.4. Histopathological Features Compared with Non-CVID Patients

To investigate in depth the clinical features of CVID patients and the biological relevance of the above described histopathological findings, we compared our results with those obtained from a control group of non-CVID patients (*n* = 21) diagnosed with early stage gastric cancer [pT1a (*n* = 15/21; 71.4%) and pT1b (*n* = 6/21; 28.6%)]. The results are presented in Table 1.

Gastric cancer in CVID patients was diagnosed at a younger age compared with non-CVID patients (median age: 43y versus 75y; Table 1; *p* < 0.001). History of *H. pylori* infection was higher within the CVID cohort (*n* = 8/9; 88.9% *versus n* = 6/14; 44.9%; Table 1; *p* = 0.036).

Regarding the histopathological characteristics of non-neoplastic mucosa, the comparison between CVID and non-CVID patients showed that lymphocytic gastritis is a distinctive characteristic of CVID patients (*n* = 7/7; 100.0% versus *n* = 1/21; 4.8%; Table 1; *p* < 0.001). Lymphoid follicles were less prominent in CVID patients when compared with non-CVID patients (*n* = 4/7; 57.1% *versus n* = 21/21; 100.0%; Table 1; *p* = 0.011). Regarding gastric cancer histotype, the comparison between the two groups showed no significant differences regarding WHO and Laurén classification (*p* = 0.089 and *p* = 0.502, respectively).

### 3.2. EBV and CMV Were Not Detected the in Non-Neoplastic Mucosa or Gastric Cancer in CVID Patients

We searched for EBV infection by EBER-ISH and CMV infection by IHC both in CVID patients and in the non-CVID control group (Table 1).

None of CVID cases showed EBER-ISH positivity in gastric cancer cells. One gastric cancer from the control group (*n* = 1/21; 4.8%) exhibited diffuse positivity. This case showed prominent lymphoid infiltration in the tumour stroma and abundant intratumoural lymphocytes, fulfilling the criteria for the diagnosis of gastric cancer with lymphoid stroma (GCLS) [30]. In non-neoplastic mucosa, scattered EBER-ISH positive lymphocytes were found in one case from the CVID patient cohort (n = 1/7; 14.3%). This is a non-specific finding and scattered EBER-ISH positive lymphocytes were also found in non-neoplastic mucosa from non-CVID patients (*n* = 15/21; 71%).

No CMV immunoreactivity was observed either in non-neoplastic mucosa or gastric cancer in any of the CVID and non-CVID patients.

### 3.3. Immune Microenvironment Changes in CVID Patients Reflect Dysfunctional Immune Microenvironment

To explore the putative immune mechanisms that may increase the susceptibility of CVID patients to gastric cancer, we analysed the density and quality of the immune infiltration in non-neoplastic mucosa (“distant from” and “adjacent to”) and tumoural tissue from CVID patients and compared the results with those obtained from the gastric specimens of non-CVID patients. We aimed to provide a spatial/temporal model to explain the role of the immune infiltrate in gastric pathogenesis in the context of CVID. Using immunohistochemistry and subsequent analysis through digital automatic quantification, we explored the infiltration rate of cytotoxic CD8+ T lymphocytes, CD4+ helper T cells, Foxp3+ regulatory T cells, GATA3+ T lymphocytes, as well as CD20+ B lymphocytes and CD138+ plasma cells.

CVID cases showed statistically significant modifications in the immune microenvironment when compared with the results obtained in non-CVID patients (Table 2 and Table 3).

**Figure 3:**
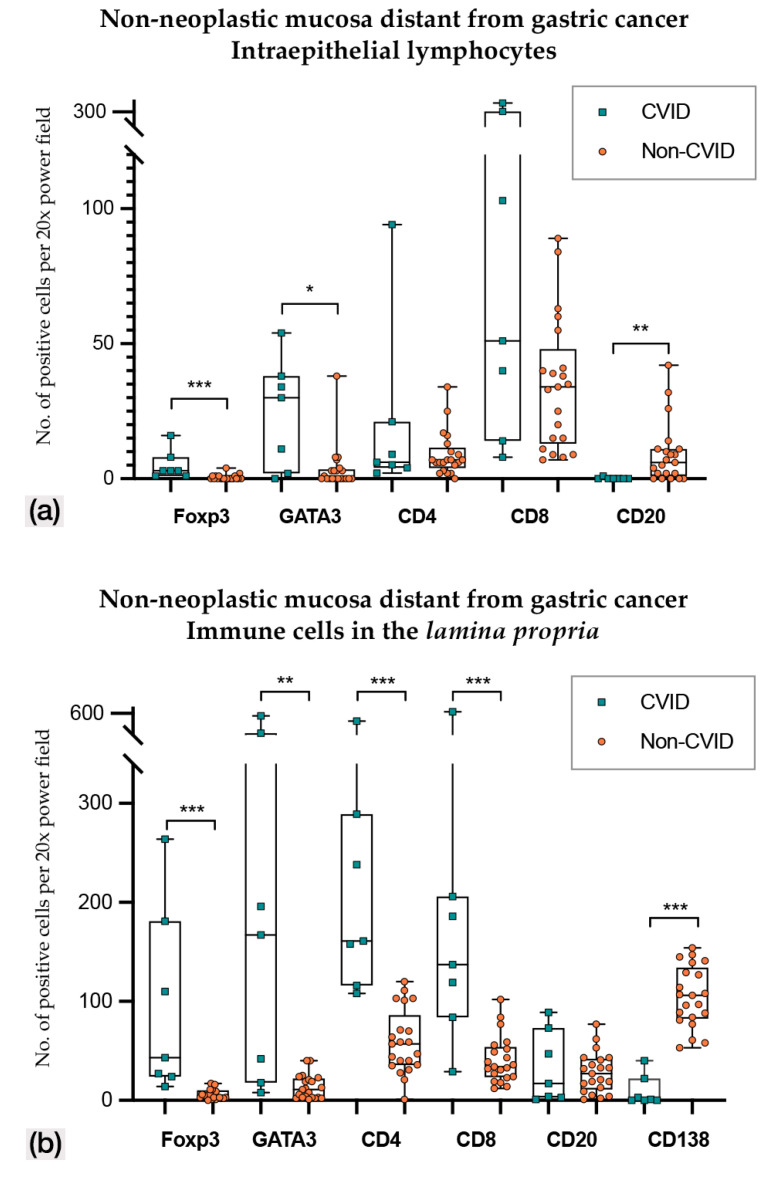
(**a**) Intraepithelial lymphocyte counts in non-neoplastic mucosa distant from gastric cancer in CVID and non-CVID patients; (**b**) counts of immune cells in the lamina propria in non-neoplastic mucosa distant from gastric cancer in CVID and non-CVID patients. Statistically significant results are highlighted by asterisks: * *p* ≤ 0.05; ** *p* ≤ 0.01; *** *p* ≤ 0.001.

**Figure 4:**
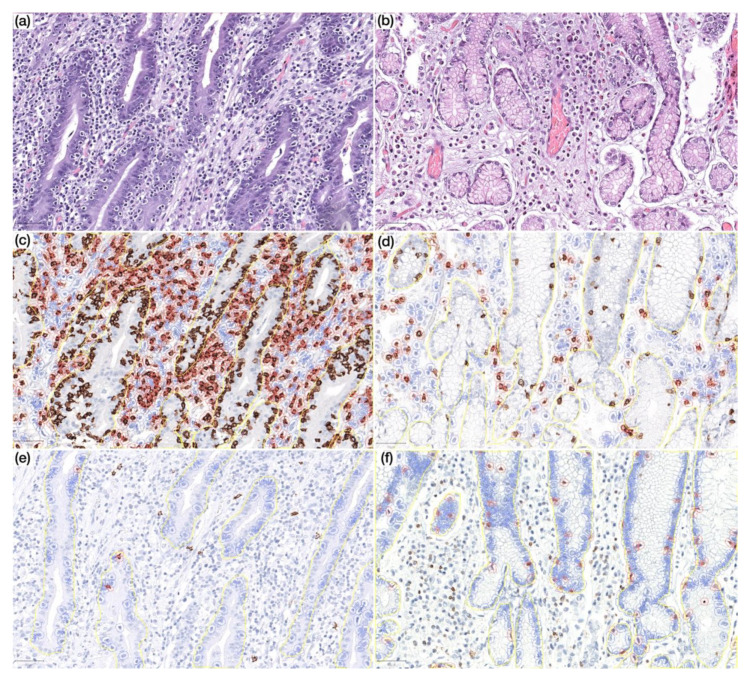
(**a**) Prominent lymphocytic gastritis in non-neoplastic mucosa distant from gastric cancer in a CVID patient (HE, 200×); (**b**) non-neoplastic mucosa distant from gastric cancer in a non-CVID patient without features of lymphocytic gastritis (HE, 200×); (**c,d**) non-neoplastic mucosa distant from gastric cancer in a CVID patient (**c**) showing higher CD8+ lymphocyte count in lamina propria as compared to a non-CVID patient (**d**) (IHC, 200×); (**e,f**) non-neoplastic mucosa distant from gastric cancer in a CVID patient (**e**) showing lower CD20+ lymphocyte count in the intraepithelial compartment as compared to a non-CVID patient (**f**) (IHC, 200×). Positive cells are highlighted by a red circle.

In non-neoplastic mucosa distant from gastric cancer (Figure 3 and Figure 4; Table 2 and Table 3), CVID cases harboured higher Foxp3+, GATA3+ and PD-L1+ intraepithelial lymphocytes (*p* < 0.001; *p* = 0.014; *p* = 0.011). Lamina propria of CVID cases showed an enrichment of Foxp3+, GATA3+, CD4+ and CD8+ lymphocytes (*p* < 0.001; *p* = 0.002; *p* < 0.001; *p* = 0.001) and higher expression of PD-L1+ immune cells (*p* = 0.011). As expected, CVID patients featured lower numbers of CD20+ intraepithelial lymphocytes (*p* = 0.003) and plasma cells (*p* < 0.001) in lamina propria as compared to non-CVID patients.

**Figure 5:**
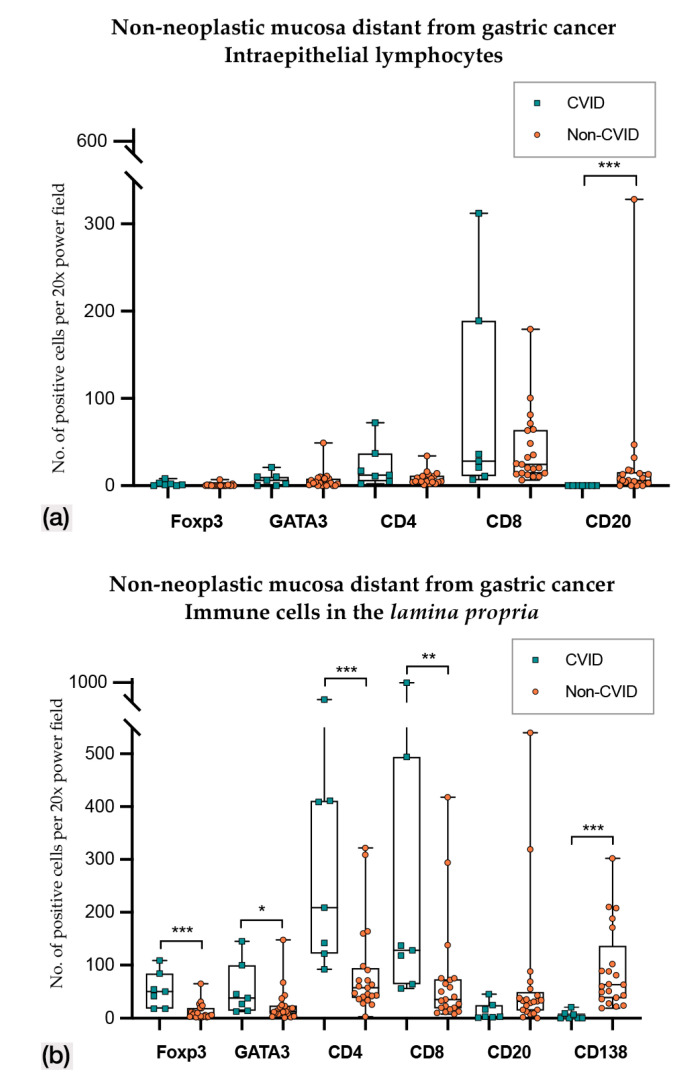
(**a**) Intraepithelial lymphocyte counts in non-neoplastic mucosa adjacent to gastric cancer from CVID and non-CVID patients; (**b**) counts of immune cells in the lamina propria in non-neoplastic mucosa adjacent to gastric cancer from CVID and non-CVID patients. Statistically significant results are highlighted by asterisks: * *p* ≤ 0.05; ** *p* ≤ 0.01; *** *p* ≤ 0.001.

In non-neoplastic mucosa adjacent to gastric cancer (Figure 5; Table 2 and Table 3), CVID patients harboured higher PD-L1+ intraepithelial lymphocytes (*p* = 0.038), while CD20+ intraepithelial lymphocytes were lower in number (*p* = 0.001); the lamina propria of CVID patients showed an enrichment of Foxp3+, GATA3, CD4+ and CD8+ lymphocytes (*p* = 0.001; *p* = 0.023; *p* = 0.001; *p* = 0.007), and there was the expected paucity of plasma cells (*p* < 0.001).

The comparison of the immune cell infiltrate in precursor adenomatous lesions (CVID: *n* = 2; non-CVID: *n* = 5) showed no significant differences (Figure S1), although this comparison is limited by the small number of cases.

**Figure 6:**
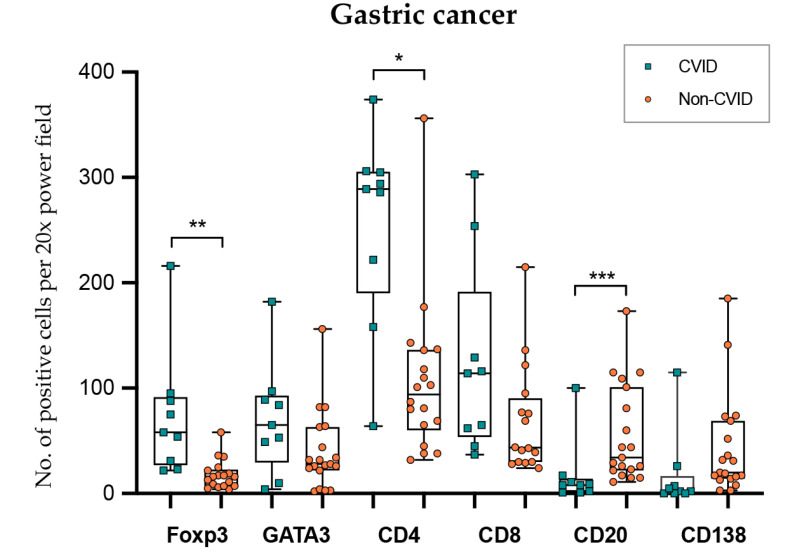
Lymphocyte counts in gastric cancer from CVID and non-CVID patients. Statistically significant results are highlighted by asterisks: * *p* ≤ 0.05; ** *p* ≤ 0.01; *** *p* ≤ 0.001.

In gastric cancer (Figure 6; Table 2 and Table 3), intraepithelial and stromal immune cells were not evaluated independently due to poor reproducibility in the recognition of the two compartments. CVID patients harboured more abundant Foxp3+ and CD4+ lymphocytes (*p* = 0.002; *p* = 0.040), while CD20+ lymphocytes were less abundant (*p* < 0.001). GATA3+ and CD8+ lymphocyte counts did not show statistical differences when the two groups were compared (*p* = 0.178; *p* = 0.689).

We explored the influence of patient age at diagnosis of gastric cancer in the non-CVID control group and we found that immune cells counts were not influenced by this parameter.

#### 3.3.1. Continuity of Immune Disturbances along Non-Neoplastic Mucosa and Gastric Cancer in CVID 

**Figure 7:**
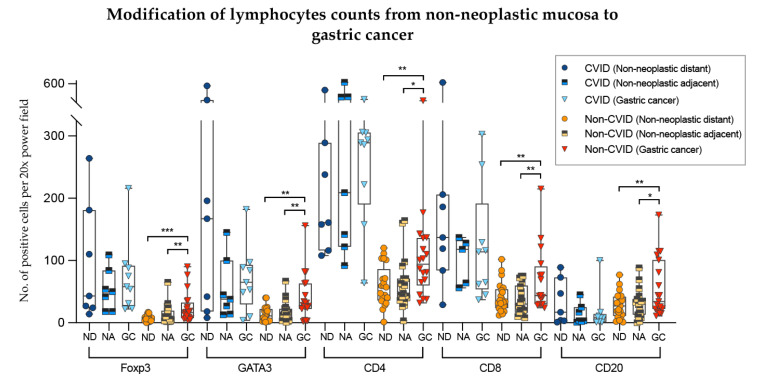
Modifications of lymphocyte counts from non-neoplastic mucosa to gastric cancer in CVID and non-CVID patients analysed separately. ND, non-neoplastic distant from tumour; NA, non-neoplastic adjacent to tumour; GC, gastric cancer. Statistically significant results are highlighted by asterisks: * *p* ≤ 0.05; ** *p* ≤ 0.01; *** *p* ≤ 0.001.

To analyse further the differences of the results obtained in the previous sections, we compared lymphocyte counts in non-neoplastic mucosa (distant from and adjacent to gastric cancer) with those from adenocarcinomas, in the two cohorts separately (Figure 7).

In non-CVID patients, there was an increase of Foxp3+, GATA3+, CD4+ and CD8+ T cell counts as well CD20+ B cell counts when the non-neoplastic mucosa distant from tumour was compared to gastric cancer (*p* = 0.001; *p* = 0.002; *p* = 0.006; *p* = 0.003; *p* = 0.004, respectively). Similar results were obtained when the non-neoplastic mucosa adjacent to tumour was compared to gastric cancer (*p values*: *p* = 0.002; *p* = 0.009; *p* = 0.010; *p* = 0.001; *p* = 0.050, respectively).

In contrast, in CVID patients, Foxp3+, GATA3+, CD4+ and CD8+ T cell counts and CD20+ B cell counts did not differ significantly when the non-neoplastic mucosa was compared to gastric cancer (*p* > 0.05).

We observed an increase of CD8+/GATA3+ lymphocytes of gastric cancers compared to non-neoplastic mucosa in non-CVID patients along with a lack of significant difference in immune cell counts in CVID-patients.

#### 3.3.2. *H. pylori* Infection May Influence the Immune Microenvironment in CVID Patients.

We compared the immune cell counts in the CVID and non-CVID patients with history of *H. pylori* infection. The detailed results are presented in Supplementary Table S3.

When *H. pylori*-infected CVID and non-CVID patients were compared, we obtained similar results in most lymphocyte count comparisons. However, in gastric cancer, *H. pylori*-infected CVID patients harboured more abundant GATA3+ and CD8+ T cells in comparison with *H. pylori*-infected non-CVID patients (*p* = 0.008; *p* = 0.005), while no significant differences were observed in the whole series (Section 3.3). Foxp3+ and CD4+ T cell counts were also increased in gastric cancer in CVID patients (*p* = 0.001; *p* = 0.003), in this case in keeping with the results obtained in the whole series (Section 3.3). Notably, in non-neoplastic mucosa adjacent to gastric cancer, GATA3+ T cell counts in the lamina propria were similar between CVID (n = 8) and non-CVID (*n* = 6) patients with a history of *H. pylori* infection (*p* = 0.065).

## 4. Discussion

Gastric cancer is an emerging phenotype within the spectrum of malignancies that affect patients with CVID, ranking second among CVID-associated cancers and first among causes of cancer-related death [16,17]. In our study, gastric cancer was not only the first cause of cancer-related death, but also the first most common malignancy in CVID patients, probably reflecting the heterogeneity of gastric cancer geographical distribution [18]. Moreover, as previously reported [17], CVID patients had early-onset gastric cancer, similarly to what happens in gastric cancer patients harbouring hereditary gastric cancer-associated syndromes [31]. This age anticipation may be explained by the permissive environment for cancer development in an immune-defective background. Additionally, the early age of gastric cancer onset in our cohort may also be explained by the rigorous endoscopic follow-up performed in our centre.

The majority of CVID patients in this cohort were diagnosed with early-stage gastric cancer (66.7%), and none of these patients died due to cancer-related causes during the follow-up period. In contrast, two patients were diagnosed with unresectable disease and died within one year due to cancer progression. Indeed, the overall survival for gastric cancer patients with unresectable/metastatic disease is less than one year, even with chemotherapy and the best supportive care [32]. All together these findings reinforce the importance of upper endoscopic surveillance in this population [22].

The dysfunctionality of the immune system in CVID patients encompasses defective B-cell activity and differentiation into plasma cells, as well as the presence of an aberrant and persistent immune activation, which may favour autoimmune disorders [33]. Worthy of note, the presence of an autoimmune background seems to be relevant for the development of malignancies in this population, as the incidence of autoimmune disorders in CVID patients with cancer has been reported to be much higher than that of CVID patients who do not develop malignancies [16]. In our series, the majority of gastric cancer CVID patients had a wide spectrum of autoimmune disorders (66.7%), pointing to the importance of further exploring the possible contribution of autoimmunity to gastric pathology and carcinogenesis in CVID. Within this context, it is relevant to emphasise that non-neoplastic mucosa in all CVID patients exhibited lymphocytic gastritis which may be a phenotypic expression of a higher propensity of the immune system to trigger an autoimmune response against gastric mucosa.

The automated digital counts of immune cells in this study provided, for the first time, a detailed characterisation of the immune dysfunctionality in the gastric mucosa of CVID patients. In keeping with a status of T cell anergy and exhaustion of the immune system, the comparison between CVID and non-CVID samples showed higher counts of Foxp3+ lymphocytes and PD-L1+ immune cells both in the intraepithelial and lamina propria compartments of CVID-associated gastric mucosa. We observed also higher GATA3+ lymphocyte counts in the gastric mucosa of CVID patients. This finding may reflect the abundancy in our samples of Foxp3+ cells, which also express GATA3 [34], as well as cytotoxic T cell dysfunctionality. Indeed, GATA3 expression by cytotoxic T lymphocytes has been described as a mechanism of T cell exhaustion, particularly in autoimmune conditions [35,36], as well as a mechanism of tumour escape from the immune surveillance [37]. Moreover, it should be emphasised that the high counts of GATA3+ lymphocytes in gastric mucosa may be the consequence of a IL-4 driven T-helper-2-cell differentiation of CD4+ T cells [38], a mechanism described in the context of *H. pylori* infection and that could influence gastric cancer pathogenesis [39,40]. This interpretation is in line with the high prevalence of *H. pylori* infection in our cohort of CVID patients (88.9%), as well as with the different results obtained comparing exclusively *H. pylori*-infected CVID and non-CVID patients. Specifically, we observed that, in non-neoplastic mucosa adjacent to gastric cancer, GATA3+ T cell counts were similar in CVID and non-CVID patients with *H. pylori* infection. Therefore, the high GATA3+ lymphocyte counts in CVID patients could be driven to some extent by *H. pylori* infection. However, the number of available cases for this comparison was limited to only 8 and 6 cases, respectively in CVID and non-CVID patients, and further data will be needed to confirm our results.

In keeping with CVID-related defects in B-cell differentiation, CD138+ plasma cells and CD20+ B lymphocytes showed lower counts both in non-neoplastic mucosa and gastric cancer in CVID patients. In agreement, lymphoid follicles were also less prominent in non-neoplastic gastric mucosa of CVID patients and the majority lacked germinal centres. Interestingly, there was consistency between the finding of circulating plasma cells (CD38^bright^IgM^negative^) and the identification of plasma cells in non-neoplastic mucosa of CVID patients.

One possible explanation for the higher gastric cancer risk in CVID is the impaired antibody responses. The risk of gastric cancer in immunodeficiencies characterised by hypogammaglobulinemia needs to be further elucidated. A recent study demonstrated that gastric cancer risk is not significantly increased in patients with IgA deficiency [15]. Isolated case reports of gastric cancer have been reported in young patients with X-linked hypogammaglobulinaemia [41,42,43,44], but the increase of gastric cancer risk in this population has not been demonstrated yet. Abnormalities in the innate immune system and T cells, rather than immunoglobulin deficiencies, could be relevant for gastric cancer pathogenesis in CVID patients.

In this study, we addressed in depth the characterisation of the immune microenvironment in CVID-associated gastric cancer, as compared with a control group of gastric cancer patients without primary immunodeficiency. The most interesting findings came up when we explored the changes between non-neoplastic mucosa and gastric cancer in the two groups. CD8+ and GATA3+ T cell counts were higher in non-neoplastic mucosa of CVID patients. However, CD8+ and GATA3+ T cells counts did not differ in gastric cancer when we compared the two groups. These findings could be due either to a decrease of CD8+/GATA3+ T cell counts in CVID patients and/or an increase of CD8+/GATA3+ T cell counts in non-CVID patients. To explore these possibilities, we analysed separately the modifications that occurred between the non-neoplastic mucosa and gastric cancer within each of the two cohorts. Our results suggest that the discordant findings encountered in non-neoplastic mucosa and gastric cancer, when comparing the two groups, are determined by an increase of CD8+ and GATA3+ T cells in gastric cancer of non-CVID patients, contrasting with the lack of significant alterations of immune cell counts in CVID patients. Therefore, changes in the immune response to carcinogenesis in CVID and non-CVID patients are not equivalent, reflecting the dysfunctionality of the CVID-associated immune system and the inability to mount an effective response to gastric cancer-driven stimuli.

Finally, we explored immune cell counts in gastric cancer by comparing exclusively *H. pylori*-infected CVID and non-CVID patients. This analysis lead to the observation that, in the context of *H. pylori* infection, CVID-associated gastric cancer harboured more abundant GATA3+ and CD8+ T cells in comparison with non-CVID gastric cancer, as observed in non-neoplastic mucosa in the whole series. These findings may reflect the importance of *H. pylori* infection in CVID-associated gastric carcinogenesis, as the presence of this particular bacterium may trigger an additional stimulation of the immune system, overcoming the intrinsic immune impairment in CVID patients. As mentioned above, the limited number of cases in this study weakens the relevance of our findings and further data are needed to explore these results. Nevertheless, several studies have highlighted the putative role of *H. pylori* infection in CVID-associated gastric carcinogenesis [20]. Moreover, the frequent and prolonged use of proton-pump inhibitors in the CVID patients of our study could have enhanced gastric carcinogenesis by promoting gastric dysbiosis [45,46].

**Figure 8:**
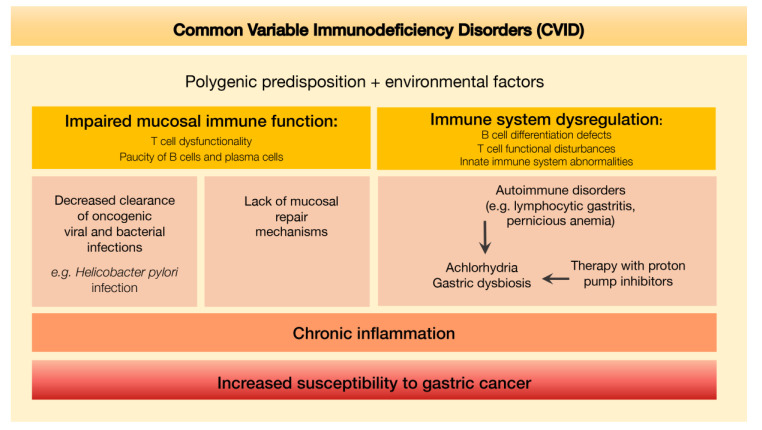
Defective immune environment in CVID and putative mechanisms leading to increased susceptibility to gastric cancer.

An integrative scheme depicting the putative mechanisms leading to increased susceptibility to gastric cancer determined by the defective immune environment associated with CVID is shown in Figure 8.

A limitation of our study is the small number of CVID-associated gastric cancers, in keeping with the rarity of this disorder. In the literature, only 59 cases have been described so far [16]. Our results could have been influenced by the young age of the CVID patients, a hypothesis that we excluded by showing, in the non-CVID control group, that the immune cells counts were not influenced by the age of the patients.

## 5. Conclusions

The study herein reported represents, to the best of our knowledge, the first in depth analysis of the immune microenvironment in gastric cancers (and respective non-neoplastic mucosa) developed in CVID patients in comparison with non-CVID patients. Our findings point to the role of the dysfunctional immune system in CVID in the development of gastric cancer.

## Figures and Tables

**Table 1:** Clinicopathological characteristics of CVID patients with gastric cancer in comparison with a control group of non-CVID patients with early stage gastric cancer.

Feature	CVID Patients *n* = 9	Non-CVID Patients *n* = 21	*p* Value
	*Median value (range)*	*Median value (range)*	
**Age**	43 (27–62)	75 (45–84)	0.000 *
	*N (%)*	*N (%)*	
**Gender** Female Male	5/9 (55.6%) 4/9 (44.4%)	11/21 (52.4%) 10/21 (47.6%)	1.000
***Helicobacter pylori*** **infection** Positive Negative	8/9 (88.9%) 1/9 (11.1%)	6/14 (42.9%) 8/14 (57.1%)	0.036 *
**Histopathological Findings in Non-Neoplastic Mucosa**			
**Lymphocitic gastritis** Present Absent NA	7/7 (100.0%) 0/7 (0.0%) 2	1/21 (4.8%) 20/21 (95.2%) 0	0.000 *
**Lymphoid follicles** Present Absent NA	4/7 (57.1%) 3/7 (42.9%) 2	21/21 (100.0%) 0/21 (0.0%) 0	0.011 *
**Neutrophilic activity** Present Absent NA	2/7 (28.6%) 5/7 (71.4%) 2	0/21 (0.0%) 21/21 (100%) 0	0.056
**Intestinal metaplasia** Present Absent NA	7/7 (100.0%) 0/7 (0.0%) 2	21/21 (100.0%) 0/21 (0.0%) 0	NA
**Histopathological features of gastric cancer**			
**pT stage (AJCC 8^th^ Ed)** [25] pT1a pT1b pT2 NA	4/7 (57.1%) 2/7 (28.6%) 1/7 (14.3) 2	15/21 (71.4%) 6/21 (28.6%) 0/21 0	0.21
**Laurén classification** [23] Intestinal Diffuse Mixed Indeterminate	6/9 (66.7%) 1/9 (11.1%) 0/9 (0.0%) 2/9 (22.2%)	13/21 (61.9%) 1/21 (4.8%) 4/21 (19.0%) 3/21 (14.3%)	0.502
**WHO classification (2019)** [24] Tubular/papillary Poorly cohesive Mucinous Mixed GCLS	6/9 (66.7%) 1/9 (11.1%) 2/9 (22.2%) 0/9 (0.0%) 0/9 (0.0%)	13/21 (61.9%) 1/21 (4.8%) 0/21 (0.0%) 4/21 (19.0%) 3/21 (14.3%)	0.089
**Precursor lesions** Present (adenoma) Absent NA	2/7 (28.6%) 5/7 (71.4%) 2	5/21 (23.8%) 16/21 (76.2%) 0	1.000
**EBV and CMV infection**			
**EBV infection (EBER-ISH)** Positive Negative	0/9 (0.0%) 9/9 (100.0%)	1/21 (4.8%) 20/21 (95.2%)	1.000
**CMV infection (IHC)** Positive Negative	0/9 (0.0%) 9/9 (100.0%)	0/21 (0.0%) 21/21 (100.0%)	NA

NA, not available; GCLS, gastric cancer with lymphoid stroma; EBV, Epstein-Barr virus; CMV, cytomegalovirus; EBER-ISH, EBV encoded RNA in situ hybridization; IHC, immunohistochemistry. Statistically significant results are highlighted by an asterisk (*).

**Table 2:** Immune cell infiltration (Foxp3, GATA3, CD4, CD8, CD20, CD138) in intraepithelial and stromal compartments of non-neoplastic mucosa (“distant from” and “adjacent to” gastric cancer) and in gastric cancer of CVID cases in comparison with non-CVID cases.

Immune Cell Biomarker	CVID Patients *n* = 9	Non-CVID Patients *n* = 21	*p* Value
1. Non-neoplastic mucosa distant from gastric cancer (intraepithelial)			
	*n* = 7/9 Median value (95% CI) (No. positive cells per 20× PF)	*n* = 21/21 Median value (95% CI) (No positive cells per 20× PF)	
Foxp3	**3.0 (1.0–8.0)**	0.0 (0.0–1.0)	0.000 *
GATA3	**30.0 (2.1–43.9)**	0.0 (0.0–6.9)	0.014 *
CD4	6.0 (2.1–21.0)	7.0 (5.0–10.0)	0.917
CD8	51.0 (11.1–303.0)	34.0 (15.0–41.0)	0.172
CD20	0.0 (0.0–0.5)	**6.0 (2.0–11.0)**	0.003 *
2. Non-neoplastic mucosa distant from gastric cancer (lamina propria)			
	*n* = 7/9 Median value (95% CI) (No. positive cells per 20× PF)	*n* = 21/21 Median value (95% CI) (No. positive cells per 20× PF)	
Foxp3	**43.0 (24.0–262.6)**	5.0 (2.0–8.0)	0.000 *
GATA3	**167.0 (18.4–570.0)**	11.0 (4.0–23.5)	0.002 *
CD4	**161.0 (108.3–289.0)**	57.0 (36.1–69.8)	0.000 *
CD8	**137.0 (84.0–206.0)**	34.0 (24.1–48.9)	0.001 *
CD20	17.0 (1.1–73.0)	27.0 (17.0–33.0)	0.876
CD138	1.0 (0.0–22.0)	**106.0 (84.1–139.6)**	0.000 *
3. Non-neoplastic mucosa adjacent to gastric cancer (intraepithelial)			
	*n* = 7/9 Median value (95% CI) (No. positive cells per 20× PF)	*n* = 21/21 Median value (95% CI) (No. positive cells per 20× PF)	
Foxp3	1.0 (0.0–4.4)	0.0 (0.0–0.0)	0.172
GATA3	6.0 (0.0–15.3)	3.0 (1.0–6.0)	0.604
CD4	12.0 (5.0–37.0)	5.0 (4.0–11.4)	0.113
CD8	28.0 (11.0–248.4)	24.0 (16.5–48.0)	0.678
CD20	0.0 (0.0–0.0)	**6.0 (3.0–13.0)**	0.001 *
4. Non-neoplastic mucosa adjacent to gastric cancer (lamina propria)			
	*n* = 7/9 Median value (95% CI) (No. positive cells per 20× PF)	*n* = 21/21 Median value (95% CI) (No. positive cells per 20× PF)	
Foxp3	**50.0 (18.0–108.2)**	6.0 (4.0–19.8)	0.001 *
GATA3	**38.0 (14.0–143.5)**	13.0 (11.0–23.0)	0.023 *
CD4	**209.0 (117.2–615.9)**	57.0 (42.0–90.3)	0.001 *
CD8	**128.0 (64.0–739.9)**	35.0 (20.1–71.8)	0.007 *
CD20	3.0 (2.0–25.0)	32.0 (20.1–44.8)	0.055
CD138	0.0 (0.0–10.5)	**63.0 (41.2–89.0)**	0.000 *
5. Gastric cancer (intraepithelial and stromal compartments)			
	*n* = 9/9 Median value (95% CI) (No. positive cells per 20× PF)	*n* = 21/21 Median value (95% CI) (No. positive cells per 20× PF)	
Foxp3	**58.0 (23.1–88.0)**	16.5 (6.6–25.0)	0.002 *
GATA3	65.0 (11.4–90.4)	30.0 (15.3–34.0)	0.178
CD4	**289.0 (222.0–305.0)**	102.0 (69.3–137.0)	0.040 *
CD8	114.0 (45.0–254.0)	72.5 (39.1–122.0)	0.689
CD20	8.0 (1.0–17.0)	**39.0 23.1–99.6)**	0.000 *
CD138	2.0 (0.0–25.6)	**31.0 (15.0–68.4)**	0.003 *

* Statistically significant results are highlighted by an asterisk (*).

**Table 3:** PD-L1 expression in non-neoplastic mucosa (“distant from” and “adjacent to” tumour) and adenocarcinomas of CVID patients in comparison with non-CVID patients.

Feature	CVID Patients	Non-CVID Patients	*p* Value
1. Non-neoplastic mucosa distant from gastric cancer (intraepithelial)			
	*n* = 7/9	*n* = 21/21	
Positive intraepithelial lymphocytes Negative	**3/7 (42.9%)** 4/7 (57.1%)	0/21 (0.0%) 21/21 (100.0%)	0.011 *
2. Non-neoplastic mucosa distant from gastric cancer (lamina propria)			
	*n* = 7/9	*n* = 21/21	
<10% positive immune cells ≥10% positive immune cells	4/7 (57.1%) **3/7 (42.9%)**	21/21 (100.0%) 0/21 (0.0%)	0.011 *
3. Non-neoplastic mucosa adjacent to gastric cancer (intraepithelial)			
	*n* = 7/9	*n* = 21/21	
Positive intraepithelial lymphocytes Negative	**3/7 (42.9%)** 4/7 (57.1%)	1/21 (4.8%) 20/21 (95.2%)	0.038 *
4. Non-neoplastic mucosa adjacent to gastric cancer (lamina propria)			
	*n* = 7/9	*n* = 21/21	
<10% positive immune cells ≥10% positive immune cells	5/7 (71.4%) 2/7 (28.6 %)	21/21 (100.0%) 0/21 (0.0%)	0.056
5. Gastric cancer (combined positive score) [27]			
	*n* = 9/9	*n* = 21/21	
<1% 1–50% >50%	2 (22.2%) 7 (77.8%) 0 (0.0%)	10 (47.6%) 8 (38.1%) 3 (14.3%)	0.117

Statistically significant results are highlighted by an asterisk (*).

## Supplementary Materials

The following are available online at https://www.mdpi.com/2073-4409/9/6/1498/s1, Table S1: Detailed IHC protocol for the antibodies used in this study, Table S2: Detailed clinico-pathological characteristics of CVID patient cohort, Table S3: Detailed results of the immune cell counting (Foxp3, GATA3, CD4, CD8, CD20) in non-neoplastic mucosa (distant from and adjacent to tumour) and adenocarcinomas of CVID patients in comparison with non-CVID patients infected by *Helicobacter pylori*. Figure S1: Immune cell counting in epithelial and lamina propria compartments in adenomatous lesions from CVID and non-CVID patients. No statistically significant differences were found when CVID and non-CVID patients were compared.
