# Supplementary material for: The Dysfunctional Immune System in Common Variable Immunodeficiency Increases the Susceptibility to Gastric Cancer
Source: Cells. 2020 Jun 19;9(6):1498. doi: 10.3390/cells9061498 (PMC7349552; doi:10.3390/cells9061498)
Supplement: Supplementary file 1 [file cells-09-01498-s001.pdf]

Appendix A

Table A1. Detailed IHC protocol for the antibodies used in this study.

Antibody	Clone	Dilution	Antigen retrieval	Incubation time (min)	Incubation tempetaure	Source
CMV	CCH2+DDG9	1:1000	Protease3-8 min + CC1-24 min-97ºC	40 min	37ºC	DAKO
CD4	SP35	RTU	CC1-32 min-95º	24 min	36ºC	Ventana - Roche
CD8	SP57	RTU	CC1-32 min-95º	28 min	37ºC	Ventana - Roche
CD8	L26	RTU	CC1-24 min-95º	24 min	37ºC	Ventana - Roche
CD20	B-A38	RTU	CC1-40 min-95º	32 min	37ºC	Cell Marque
CD138/Syndecan-1	AB54501	1:100	CC1-40 min-95º	36 min	37ºC	Abcam
Foxp3	D13C9	1:200	CC1-64 min-95º	60 min	36ºC	Cell signaling
GATA3	22C3	1:80	CC1-64 min-95º	36 min	37ºC	DAKO
PD-L1	CCH2+DDG9	1:1000	Protease3-8 min + CC1-24 min-97ºC	40 min	37ºC	DAKO

RTU, Ready To Use.

Table A2. Detailed clinico-pathological characteristics of CVID patient cohort.

**Patient ID**	**1**	**2**	**3**	**4**	**5**	**6**	**7**	**8**	**9**
**Gender**	Female	Male	Female	Female	Male	Male	Female	Female	Male
**Age at first CVID symptoms**	1	17	13	16	7	2	16	NA	15
**Age at CVID diagnosis**	22	19	21	18	16	22	30	46	15
**Age at IgG replacement therapy**	25	25	28	34	29	39	30	46	15
**Age at gastric cancer diagnosis**	28	43	40	27	45	60	38	62	47
**Family history**									
Primary immunodeficiency	No	No	No	No	IgA deficiency	No	No	No	No
Autoimmune disorder	Vitiligo	No	SS, ATD	Type 1 DM, ATD	No	No	No	No	No
Malignancy in relatives (years at diagnosis)	Breast (73)<br>Prostate (80)	Colon/rectum (n=2; 80; 75)<br>**Stomach** (n=2; 68; 68)	Breast (51)<br>CNS (57)	Colon/rectum (50)<br>Breast (36)	Breast (69)	RCC (80)<br>Peritoneal carcinomatosis of unknown primary site (60)	Breast (57)	No	**Stomach** (58)
**Autoimmune disorders**	**Pernicious anaemia**<br>AIP<br>RA-like symptoms	**Pernicious anaemia**<br>Psoriasis	ITP<br>Vasculitis<br>RA-like symptoms	Alopecia<br>RA-like symptoms	**Pernicious anaemia**	No	**Pernicious anaemia**	No	No
**Granulomatous disease**	Lung/skin granulomas	GLILD	No	GLILD	GLILD	No	No	No	No
**Gastrointestinal infection (excluding *H. pylori*)**	*C. jejuni*<br>*Salmonella spp.*<br>CMV	*C. jejuni*<br>*Salmonella spp.*<br>*Giardia lamblia*<br>CMV	*C. jejuni*<br>*Salmonella spp.*<br>*Giardia lamblia*	EBV<br>Norovirus<br>CMV	*Giardia lamblia*	*C. jejuni*<br>*Giardia lamblia*	*Giardia lamblia*	*Giardia lamblia*	*C. jejuni*<br>*Giardia lamblia*

H. pylori **infection**	Positive	Positive	Positive	Positive	Positive	Positive	Positive	Positive	Negative
**Prolonged use of PPI**	Yes	Yes	Yes	NA	Yes	No	Yes	Yes	NA
**Histopathological features before GC diagnosis**	Chronic gastritis<br>LGD and HGD	Chronic gastritis<br>IM<br>LGD adenomas	Chronic gastritis<br>Hyperplastic polyp	Chronic gastritis	Chronic gastritis<br>IM	NA	Chronic gastritis<br>IM	Chronic gastritis<br>IM	NA
**Surgery**	Total gastr.	Total gastr.	Total gastr.	Total gastr.	No (unresectable disease)	No (unresectable disease)	Total gastr.	Distal gastr.	Total gastr.
**Chemotherapy**	No	No	No	No	No	Palliative	No	No	No
**Other malignancy (years at diagnosis)**	No	Rectal adenocarcinoma (49)	No	No	HCC (45)	No	No	No	No
**Histopathological findings in non-neoplastic mucosa (n=7/9)**									
**Atrophic chronic gastritis**	Present	Present	Present	Present	Present	NA	NA	Present	Present
**Lymphocytic gastritis**	Exuberant	Present	Present	Exuberant	Present	NA	NA	Present	Present
**Lymphoid follicles**	Absent	Absent	Absent	Present	Present	NA	NA	Present	Present
**Neutrophilic activity**	Absent	Absent	Absent	Present	Absent	NA	NA	Absent	Present
**Intestinal metaplasia**	Absent	Extensive	Extensive	Present	Present	NA	NA	Present	Extensive
**EBV infection (EBER-ISH)**	Absent	Absent	Absent	Absent	Rare + lymphocytes	NA	NA	Absent	Absent
**CMV infection (IHC)**	Absent	Absent	Absent	Absent	Absent	NA	NA	Absent	Absent

**Histopathological features of gastric cancer**									
**Location**	Antrum	Antrum	Corpus	Antrum	Antrum	Corpus	Corpus	Antrum	Corpus
**pT stage**	pT1a	pT1a	pT2	pT1a	NA	NA	pT1a	pT1b	pT1b
**Greatest dimension**	4.5 cm	0.5 cm	1.5 cm	1.8 cm	NA	NA	1.0 cm	2.5 cm	0.6 cm
**pN stage**	pN0	pN0	pN0	pN0	NA	NA	pN0	pN0	pN0
**Laurén classification**	Intestinal	Diffuse	Intestinal	Indeterminate	Intestinal	Intestinal	Intestinal	Indeterminate	Intestinal
**WHO classification**	Tub/Pap	Poorly cohesive	Tub/Pap	Mucinous	Tub/Pap	Tub/Pap	Tub/Pap	Mucinous	Tub/Pap
**Grading**	Low grade	NA	High grade	NA	High grade	High grade	Low grade	NA	Low grade
**Precursor lesion**	Intestinal adenoma LGD	Absent	Absent	Absent	NA	NA	Absent	Intestinal adenoma LGD	Absent
**EBV infection (EBER-ISH)**	Absent	Absent	Absent	Absent	Absent	Absent	Absent	Absent	Absent
**CMV infection (IHC)**	Absent	Absent	Absent	Absent	Absent	Absent	Absent	Absent	Absent

AIP, Autoimmune pancreatitis; **ATD**, Autoimmune Thyroid Disease; ***C. jejuni***, *Campylobacter jejuni*; **CMV**, Cytomegalovirus; **CNS**, Central Nervous System; **DM**, Diabetes Mellitus; **EBER-ISH**, EBV Enconded RNA *In Situ* Hybridization; **EBV**, Epstein-Barr Virus; **Gastr**, Gastrectomy; **GC**, Gastric Cancer; **GLILD**, Granulomatous Lymphocytic Interstitial Lung Disease; ***H. pilory***, Helicobacer pylori; **HCC**, Hepatocelular Carcinoma; **HGD**, High Grade Dysplasia; **IHC**, Immunohistochemistry; **IM**, Intestinal Metaplasia; **ITP**, Immune Thrombocytopenia; **LGD**, Low Grade Dysplasia; **NA**, Not Available; **PPI**, Proton-pump Inhibitor; **RA**, Rheumatoid Arthritis; **RCC**, Renal Cell Carcinoma; **RLH**, Reactive Lymphoid Hyperplasia; **SS**, Sjogren's syndrome; **Tub/pap**, Tubular/Papillary.

Table A3. Detailed results of the immune cell counting (Foxp3, GATA3, CD4, CD8, CD20) in non-neoplastic mucosa (distant from and adjacent to tumour) and adenocarcinomas of CVID patients in comparison with non-CVID patients infected by *Helicobacter pylori*.

**Immune cell biomarker**	**CVID patients**<br>***H. Pylori* positive**<br>**n=8**	**Non-CVID patients**<br>***H. Pylori* positive**<br>**n=6**	***p value***
1. Non-neoplastic mucosa distant from gastric cancer (intraepithelial)			
	n=6/8 *(95% CI)*<br>*Median value*<br>*(No. positive cells per 20x PF)*	n=6/6 *(95% CI)*<br>*Median value*<br>*(No. positive cells per 20x PF)*	
Foxp3	3.0 (3.0-16.0)	0.0 (0.0-1.0)	**0.015***
GATA3	32.0 (2.0-38.0)	1.5 (0.0-5.0)	**0.015***
CD4	5.5 (2.0-9.0)	11.0 (2.0-34.0)	0.818
CD8	77.0 (40.0-374.0)	36.5 (11.0-52.0)	0.132
CD20	0.0 (0.0-0.0)	6.0 (0.0-21.0)	**0.015***
2. Non-neoplastic mucosa distant from gastric cancer (lamina propria)			
	n=6/8 *(95% CI)*<br>*Median value*<br>*(No. positive cells per 20x PF)*	n=6/6 *(95% CI)*<br>*Median value*<br>*(No. positive cells per 20x PF)*	
Foxp3	68.05 (24.0-181.0)	7.0 (3.0-11.5)	**0.004***
GATA3	181.5 (18.4-570.0)	11.5 (3.0-25.0)	**0.004***
CD4	159.5 (116.0-509.0)	67.0 (2.2-101.0)	**0.004***
CD8	161.5 (84.0-186.0)	36.5 (24.0-43.0)	**0.002***
CD20	10.5 (7.5-89.0)	23.0 (14.3-5.1)	0.589
CD138	2.0 (0.0-40.0)	110.0 (61.0-141.0)	**0.002***
3. Non-neoplastic mucosa adjacent to gastric cancer (intraepithelial)			
	n=6/8 *(95% CI)*<br>*Median value*<br>*(No. positive cells per 20x PF)*	n=6/6 *(95% CI)*<br>*Median value*<br>*(No. positive cells per 20x PF)*	
Foxp3	1.0 (0.0-1.0)	0.0 (0.0-1.0)	0.310

GATA3	8.0 (0.0-21.0)	1.5 (0.0-7.0)	0.180
CD4	11.5 (3.5-37.0)	8.0 (2.1-33.3)	0.699
CD8	32.0 (21.0-189.0)	13.5 (11.0-38.5)	0.310
CD20	0.0 (0.0-0.0)	4.5 (2.0-9.4)	**0.002***
4. Non-neoplastic mucosa adjacent to gastric cancer (lamina propria)			
	n=6/8<br>*Median value (95% CI)*<br>*(No. positive cells per 20x PF)*	n=6/6<br>*Median value (95% CI)*<br>*(No. positive cells per 20x PF)*	
Foxp3	48.0 (18.0-109.0)	5.5 (2.1-10.0)	**0.002***
GATA3	36.0 (27.0-145.0)	12.0 (2.0-29.6	**0.065**
CD4	75.5 (60.0-409.0)	51.0 (3.0-97.1)	**0.004***
CD8	132.05 (128.0-494.0)	21.5 (13.2-40.0)	**0.002***
CD20	3.0 (3.0-17.0)	30.0 (8.3-37.8)	**0.026***
CD138	0.0 (0.0-0.0)	57.0 31.8-130.3)	**0.002***
5. Gastric cancer (intraepithelial and stromal compartments)			
	n=8/8<br>*Median value (95% CI)*<br>*(No. positive cells per 20x PF)*	n=6/6<br>*Median value (95% CI)*<br>*(No. positive cells per 20x PF)*	
Foxp3	66.5 (54.0-75.0)	8.0 (6.0-16.0)	**0.001***
GATA3	74.5 (53.0-65.0)	26.0 (3.0-28.0)	**0.008***
CD4	291.5 (222.0-372.0)	41.5 (32.1-101.0)	**0.003***
CD8	115.0 (45.0-303.0)	34.5 (24.2-48.7)	**0.005***
CD20	8.5 (1.0-11.0)	22.5 (15.1-51.2)	**0.029**
CD138	2.0 (2.0-26.0)	16.5 8.5-52.0)	**0.108**

Statistically significant results are highlighted by an asterisk (*). Differences in comparison with the analyses performed in the totality of CVID and non-CVID patients (Table 2) are highlighted in red.

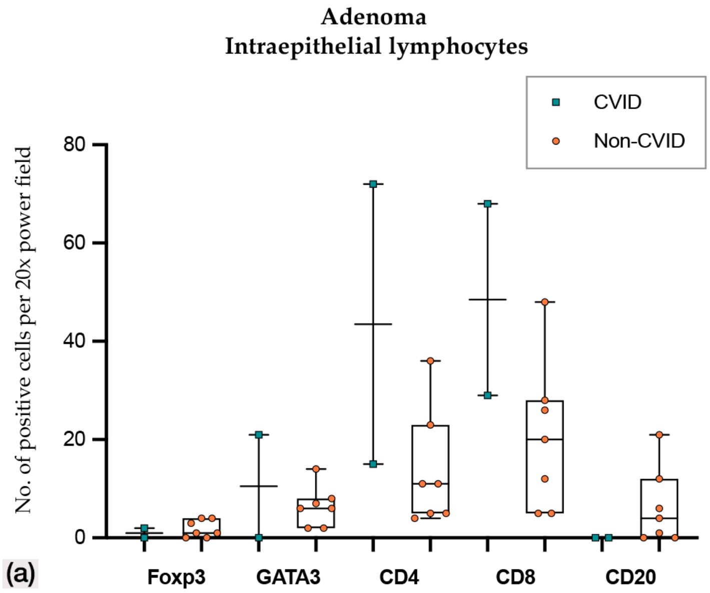

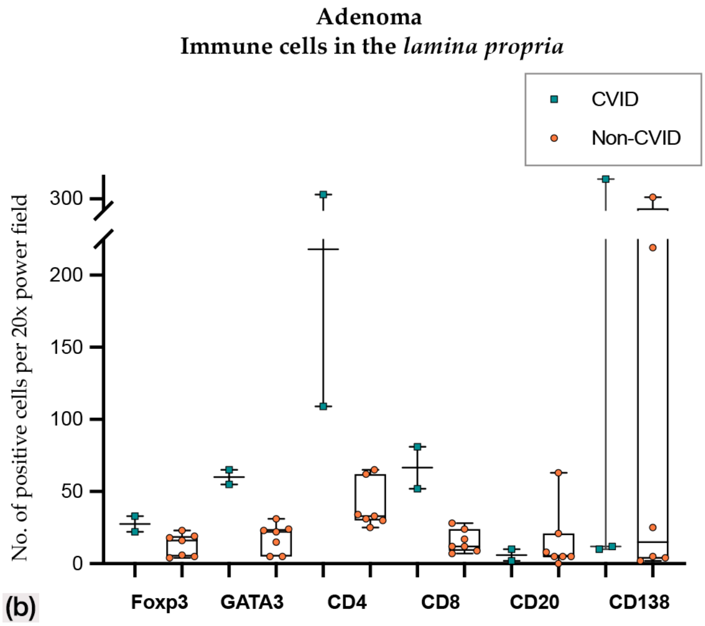

Figure A1. Immune cell counting in epithelial and lamina propria compartments in adenomatous lesions from CVID and non-CVID patients. No statistically significant differences were found when CVID and non-CVID patients were compared.
